# Outcome prediction in haematological patients requiring admission to the ICU

**DOI:** 10.1186/cc9931

**Published:** 2011-03-11

**Authors:** S Gopal, N Green, M Myint, A Jacobs

**Affiliations:** 1 Heart and Lung Centre, Wolverhampton, UK; 2 New Cross Hospital, Wolverhampton, UK

## Introduction

The outcome of haematological patients admitted to the ICU is improving [1,2]. However little is known of the predictive factors that determine hospital outcome in this group of patients. We hypothesised that certain haematological factors may predict a worse outcome in these patients requiring admission to the ICU.

## Methods

We retrospectively reviewed all haematological patients admitted to a 15-bed medicosurgical ICU of a teaching hospital over a 5-year period from 1 April 2005 to 31 March 2010. Data on validated outcome predictors including age, APACHE II score, APACHE II predicted mortality, length of ICU stay, and requirement for mechanical ventilation were collected. Furthermore outcome predictors deemed important in haematological patients were also collected, including neutropaenic status at onset of illness, malignancy status at onset of illness and whether chemotherapy was received within 30 days of admission to the ICU. We performed logistic regression analysis to model these variables against hospital mortality.

## Results

Fifty-six haematological patients were admitted to the ICU during the study period. Data from three patients were incomplete and they were therefore excluded from the analysis. Mean age (SD) 54 (18.5) years; mean APACHE II score (SD) 23.4 (6.8); mean APACHE II predicted mortality (SD) 50.6 (23.8)%; mean ICU stay (SD) 4.6 (3.7) days. Twenty patients (35.7%) were mechanically ventilated on admission to the ICU. Thirteen patients (26%) were neutropaenic at onset of critical illness; 40 patients (75.5%) had a haematological malignancy and 31 patients (56.6%) had received chemotherapy within 30 days of the onset of critical illness. The standardised mortality ratio (95% CI) for this cohort of patients was 0.86 (0.82 to 0.91). Logistic regression analysis revealed no relationship between these variables and hospital mortality even after adjusting for age, APACHE II score, length of ICU stay and requirement for mechanical ventilation. Adjusted OR (95% CI) for neutropaenic status at onset of critical illness was 1.8 (0.3 to 9.1) *P *= 0.46. Malignancy at onset of illness OR was 0.54 (0.1 to 3.7) *P *= 0.53. Chemotherapy within 30 days of admission to ICU OR was 0.4 (0.1 to 2.2) *P *= 0.30.

## Conclusions

Haematological factors including neutropaenia, haematological malignancy and recent chemotherapy do not predict worse outcomes in this group of patients. With improving mortality rates, all haematological patients should be considered for admission to the ICU.
